# Evaluation of the VISAGE Basic Tool for Appearance and Ancestry Prediction Using PowerSeq Chemistry on the MiSeq FGx System

**DOI:** 10.3390/genes11060708

**Published:** 2020-06-26

**Authors:** Leire Palencia-Madrid, Catarina Xavier, María de la Puente, Carsten Hohoff, Christopher Phillips, Manfred Kayser, Walther Parson

**Affiliations:** 1 Institute of Legal Medicine, Medical University of Innsbruck, 6020 Innsbruck, Tirol, Austria; leire.palencia@ehu.eus (L.P.-M.); catarina.gomes@i-med.ac.at (C.X.); mdelcarmendela.puente@usc.es (M.d.l.P.); 2 BIOMICs Research Group, Lascaray Research Center, University of the Basque Country, 01006 Vitoria-Gasteiz, Basque Country, Spain; 3 Forensic Genetics Unit, Institute of Forensic Sciences, University of Santiago de Compostela, 15782 Santiago de Compostela, Galicia, Spain; c.phillips@mac.com; 4 Institut für Forensische Genetik GmbH, 48161 Münster, Nordrhein-Westfalen, Germany; gednap@ifg-ms.de; 5 Department of Genetic Identification, Erasmus MC University Medical Center Rotterdam, 3015 CN Rotterdam, South Holland, The Netherlands; m.kayser@erasmusmc.nl; 6 Forensic Science Program, The Pennsylvania State University, University Park, Pennsylvania, PA 16802, USA

**Keywords:** forensic DNA phenotyping, appearance, ancestry, BGA, HIrisPlex-S, EVC prediction, phenotype prediction, DNA phenotyping, PowerSeq, VISAGE

## Abstract

The study of DNA to predict externally visible characteristics (EVCs) and the biogeographical ancestry (BGA) from unknown samples is gaining relevance in forensic genetics. Technical developments in Massively Parallel Sequencing (MPS) enable the simultaneous analysis of hundreds of DNA markers, which improves successful Forensic DNA Phenotyping (FDP). The EU-funded VISAGE (VISible Attributes through GEnomics) Consortium has developed various targeted MPS-based lab tools to apply FDP in routine forensic analyses. Here, we present an evaluation of the VISAGE Basic tool for appearance and ancestry prediction based on PowerSeq chemistry (Promega) on a MiSeq FGx System (Illumina). The panel consists of 153 single nucleotide polymorphisms (SNPs) that provide information about EVCs (41 SNPs for eye, hair and skin color from HIrisPlex-S) and continental BGA (115 SNPs; three overlap with the EVCs SNP set). The assay was evaluated for sensitivity, repeatability and genotyping concordance, as well as its performance with casework-type samples. This targeted MPS assay provided complete genotypes at all 153 SNPs down to 125 pg of input DNA and 99.67% correct genotypes at 50 pg. It was robust in terms of repeatability and concordance and provided useful results with casework-type samples. The results suggest that this MPS assay is a useful tool for basic appearance and ancestry prediction in forensic genetics for users interested in applying PowerSeq chemistry and MiSeq for this purpose.

## 1. Introduction

Forensic DNA Phenotyping (FDP) involves the prediction of the physical appearance through determining externally visible characteristics (EVCs) and the biogeographic ancestry (BGA) from DNA [1,2,3]. FDP can provide investigative leads, when standard DNA identification is not possible, for example, due to the lack of a suspect’s reference sample or a lack of matches in National DNA Databases [3,4]. Recent studies focused on fine-tuning the selection of DNA markers to predict physical appearance, e.g., [5,6,7,8,9,10,11]. The most successful studies developed models for eye, hair and skin color with promising prediction performance analyzing only a few dozen single nucleotide polymorphisms (SNPs) [12,13,14]. It has been demonstrated that the analysis of nuclear SNPs also aids the estimation of the biogeographical ancestry (BGA) by using at least thirty or more SNPs to obtain reliable results [15,16,17,18,19]. Targeted Massively Parallel Sequencing (MPS) technologies enable the simultaneous amplification of hundreds of DNA markers at low DNA input levels thus providing sensitive tools that keep sample consumption low, which has started to be applied for forensic purposes including appearance and/or ancestry prediction from DNA, e.g., [20,21,22]. The need for combining more and more SNPs for combined appearance and ancestry prediction and the suitability of targeted MPS has triggered the development of a panel consisting of 153 markers for predicting appearance and ancestry in the framework of the VISAGE (VISible Attributes through GEnomics) Consortium (http://www.visage-h2020.eu/). These markers had recently been implemented into a combined MPS tool based on Ion AmpliSeq (for Thermo Fisher platforms), a chemistry of Thermo Fisher Scientific for custom amplicon design [23]. This study presents experimental results on the evaluation of this panel based on the PowerSeq chemistry analyzed on a MiSeq FGx System (Illumina). A series of test were conducted, including sensitivity, repeatability, concordance and analysis of casework “mock” samples.

## 2. Materials and Methods

### 2.1. Samples and Experimental Design

The 2800 M Control DNA (Promega) was used for sensitivity and repeatability tests. DNA extracts provided in the course of the GEDNAP (German DNA Profiling Group; https://www.gednap.org/de/) proficiency tests were used as casework-type samples, and Coriell samples with known genotypes were analyzed for concordance experiments.

The sensitivity of the assay was tested by a serial dilution of 2800 M Control DNA from 1 ng to 10 pg in duplicates. Repeatability was tested analyzing optimum DNA input triplicates of 2800 M at 0.5 and 1 ng. For this purpose, one extra replicate of each DNA amount was added to the duplicates used for the sensitivity test. Five DNA extracts from GEDNAP proficiency tests (42-S3, 44-S3, 45-S2, 49-S4 and 53-S1) quantified via real-time qPCR [24] were analyzed using the recommended DNA input of 0.5 ng. Concordance was evaluated using four Coriell samples that have known genotypes at 0.5 ng of DNA input. The genotypes of these samples are published in the genetic databases of the 1000 Genomes Project ([25,26]; samples NA06994, NA07000 and NA18498) and the Simons Genome Diversity Project (SGDP) ([27]; sample NA11200). Additionally, one non-template control (NTC) sample was used. The 24 samples of this study were sequenced together in one run.

### 2.2. VISAGE Basic Tool for Predicting Appearance and Ancestry Using PowerSeq Chemistry

The VISAGE Basic Tool for predicting appearance and ancestry (BT A&A) was developed by the VISAGE Consortium and has recently been implemented using AmpliSeq [23]. It consists of 153 SNPs, 41 of which represent the HIrisPlex-S panel to predict eye, hair and skin color [5,12,13,14] and 115 forming the continental BGA informative panel (3 overlapping markers in both panels). In this study we implemented the BT A&A provided using PowerSeq chemistry (Promega, Madison, WI, USA) and MiSeq, which we refer to here as VISAGE BT A&A (PSeq).

### 2.3. Library Preparation and Sequencing

All samples were analyzed following the manufacturer’s recommendations [28]. A first amplification PCR was conducted including 5 µL of PowerSeq 5X Master Mix (Promega, Madison, WI, USA), 5 µL of VISAGE primer mix 5X (Promega, Madison, WI, USA), template DNA and distilled water up to the total volume of 25 µL. PCR was conducted on an Applied Biosystems GeneAmp PCR System 9700 (Thermo Fisher Scientific, Waltham, MA, USA) and the thermal cycling conditions were 1 min at 96 °C followed by 30 cycles of amplification with 10 s at 94 °C, 1 min at 60 °C and 30 s at 72 °C, to finish with a final extension step of 10 min at 60 °C.

The amplification products were purified with the ProNex Size-Selective Purification System (Promega, Madison, WI, USA) following the manufacturer’s instructions [28,29]. This was conducted adding 5 µL of 1080 µg/mL Proteinase K (Promega, Madison, WI, USA) and 105 µL of ProNex Chemistry (Promega, Madison, WI, USA) to each 25 µL of amplification product and performing two washing steps prior to the purified product elution. The KAPA Hyper Prep Kit (Kapa Biosystems, Roche Sequencing, Wilmington, MA, USA) was used for library preparation. The end repair and A-tailing reaction was conducted prior to the adapter ligation step of the Illumina TruSeq Index adapters (Illumina, San Diego, CA, USA) as indicated by the manufacturer [28,30]. The libraries were purified with the ProNex Size-Selective Purification System (Promega, Madison, WI, USA). This was conducted by adding 220 µL of ProNex Chemistry (Promega, Madison, WI, USA) to 110 µL of each library, followed by two washing steps and the elution of the purified libraries.

The purified libraries were quantified with the PowerSeq Quant MS System (Promega, Madison, WI, USA) following the manufacturers’ instructions [31]. Libraries were diluted to 4 nM, equally pooled, denatured with 0.2N NaOH and diluted to 20 pM with HT1 buffer (Illumina, San Diego, CA, USA). The final sequencing mix was prepared with 300 µL of HT1 buffer (Illumina, San Diego, CA, USA), 255 µL of pooled 20 pM denatured libraries and 45 µL of 20 pM denatured PhiX Control (Illumina, San Diego, CA, USA). Sequencing of the 24 libraries of this study was performed in one flow cell, on a MiSeq FGx with 2 × 150 cycles and the MiSeq Reagent kit V3 (Illumina, San Diego, CA, USA).

### 2.4. Data Processing

Sequences were aligned to the human genome (hg19) using the BWA-MEM algorithm [32]. Data manipulation, and BAM and BAI creation were performed using SAMtools [33] and PICARD tools. Data extraction and variant calling were performed with GATK [34,35]. The interpretation threshold for each allele was set at 20% of the total reads obtained for that locus. Sequencing data of each locus were also visualized with Integrative Genomics Viewer (IGV) v2.6.3 [36]. Microsoft Excel and RStudio v1.2.1335 [37,38] were used for data processing and statistical analyses.

## 3. Results and Discussion

### 3.1. Negative Control

The baseline noise produced with the VISAGE BT A&A (PSeq) was investigated by analyzing the NTC sample. As a result of this analysis, a total of 1502 reads were obtained in 116 SNPs (75.8% of the SNPs included in the assay) with a mean read depth per marker of 9.82 (±49.81). Only in three of the SNPs were more than 25 reads observed, two of which (rs1040934 and rs1426657) showed more than 100 reads (248 and 566 reads, respectively). Nevertheless, the mean read depth per marker obtained for the NTC was 128 times lower than the value observed for samples with 10 pg of DNA, the lowest input amount tested here. Comparable values for NTC samples have been seen in similar studies [39,40,41].

### 3.2. Sensitivity

**Figure 1:**
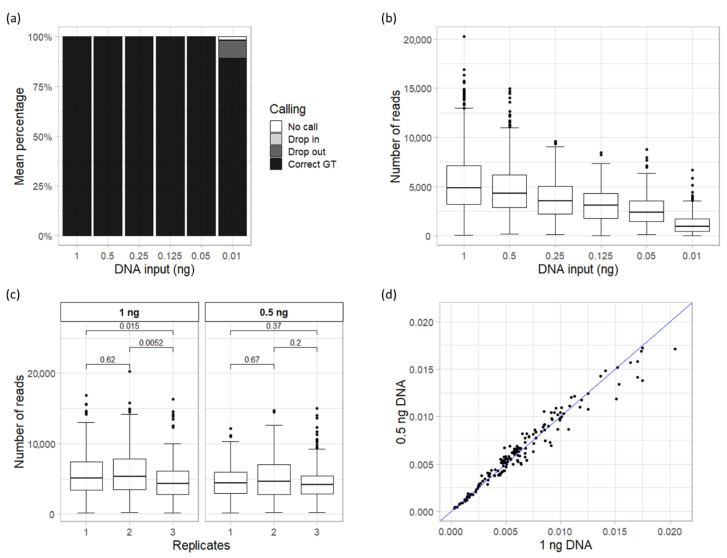
Results obtained with the VISible Attributes through GEnomics (VISAGE) Basic Tool for predicting appearance and ancestry (VISAGE BT A&A) (PSeq) assay for sensitivity and repeatability tests. (**a**) Mean percentage of correct genotypes and incorrect genotypes (drop-out, drop-in and no calls) obtained in the sensitivity test from the dilution series of 2800 M Control DNA (1 ng–10 pg) analyzed in duplicates. (**b**) Mean number of reads yielded per marker for each dilution step. (**c**) Average read depth per marker obtained in each replicate of 1 ng and 0.5 ng used for repeatability test. *p*-values of comparisons between replicates for Wilcoxon test are shown. (**d**) Mean normalized read depth per marker of 1 ng versus 0.5 ng replicates.

Decreasing amounts of 2800 M Control DNA (Promega), from 1 ng to 10 pg, were used as template DNA to test the sensitivity of the VISAGE BT A&A (PSeq). In the absence of the “true” genotypes we assumed the correct calls based on the consensus of all 2800 M analyses. The correct genotype of the complete set of 153 SNPs was obtained in all the replicates down to 125 pg input DNA (Figure 1a). Replicates with 50 pg produced 99.67% of correct genotypes with only one allele drop-out in one of the replicates. For the lowest amount of DNA template tested (10 pg) the number of correct genotypes dropped to 89.22% due to rising numbers of allele and locus drop-out in both replicates and the appearance of one allele drop-in. The effect of decreasing DNA amount is more evident in the number of sequencing reads with mean read depths per marker from 5809 ± 3572 reads at 1 ng to 1254 ± 1111 reads at 10 pg (Figure 1b).

Nonetheless, we did not observe a linear correlation between the allele drop-out and the number of reads obtained for the affected markers. A third of the loci with drop-out in replicates of 10 pg yielded more than 900 reads, while 14.7% of correctly genotyped loci had less than 300 reads. This points to unbalanced allele amplification during PCR, probably due to the low amount of template DNA [42,43].

### 3.3. Repeatability

Three replicates of 1 and 0.5 ng of template DNA were analyzed to assess the repeatability of the VISAGE BT A&A (PSeq) assay. The average total number of reads showed a reduction from 1 ng replicates (840,590 reads) to 0.5 ng replicates (729,618 reads), with a noticeably wider standard deviation (SD) in 1 ng replicates (±85,890 reads) than in 0.5 ng replicates (±31,202 reads), albeit not statistically significant. A Kruskal-Wallis test showed that there were significant differences between the average reads per marker yielded by 1 ng replicates (*p* < 0.05), but no significant differences among the 0.5 replicates (Figure 1c). A Wilcoxon test revealed that the difference between 1 ng replicates was due to the third replicate, which had significantly lower mean reads per marker than the others. This difference may be due to amplification and preparation of the libraries of the third replicates, which were performed on a different day. However, the observed differences could also represent normal variation of the assay.

Normalized read depth per marker (calculated as number of target reads per marker/total number of reads in a sample) was analyzed in the replicates of both DNA amounts. The comparison of the mean normalized read depth between 1 ng and 0.5 ng showed no clear difference between DNA inputs (Figure 1d) and Kruskal–Wallis test confirmed the absence of significant differences. The mean normalized read depth was 0.00654, and only 31 SNPs (20.26%) performed below 0.004 at both amounts of DNA. This shows that, even if the amount of input DNA had an impact on the total reads, the read distribution between markers stayed stable. Thus, the VISAGE BT A&A (PSeq) is a robust assay for input DNA of 1 and 0.5 ng, the latter being the optimum amount recommended by the manufacturer.

### 3.4. Locus, Allele Balance and Base Misincorporation Rate

**Figure 2:**
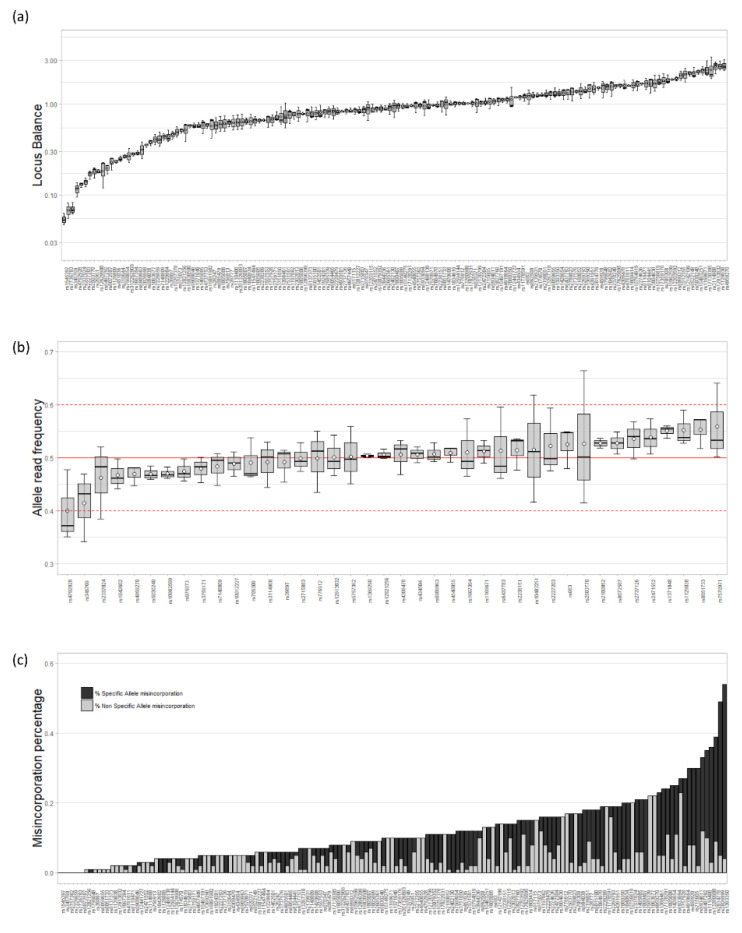
Sequencing parameters obtained with the VISAGE BT A&A (PSeq) assay from the analysis of the three replicates of 2800 M Control DNA at 0.5 ng. (**a**) Distribution of the locus balance. (**b**) Distribution of the allele read frequency of every heterozygote locus of 2800 M Control DNA. A continuous line marks the expected optimum (0.5) while the dotted lines indicate the lower and upper thresholds (0.4 and 0.6, respectively). (**c**) Distribution of base misincorporation percentage, showing the percentage of specific (SBM) and non-specific (NBM) base misincorporations for each locus.

Replicates of 0.5 ng, defined as an optimum amount of input DNA recommended by Promega, were used to assess the general performance of the VISAGE BT A&A (PSeq) assay for the analysis of diverse sequencing parameters. The performance of loci amplification and sequencing was evaluated by comparing the read depth obtained for every locus. Figure 2a shows the distribution of locus balance, calculated as the ratio between the reads obtained for each locus and the average number of reads per locus [44]. Most of the loci were well balanced, with values close to 1 (75.2%). Only 11 loci (7.2%) showed more than double the average number of reads per locus of the whole panel, and 27 loci (17.6%) performed below half of the average of reads per locus.

For heterozygote genotypes (40 SNPs) observed with the analyzed DNA samples, the allele read frequency was estimated as the number of reads obtained for the reference allele (ref) divided by the sum of reads for the reference and the alternative allele (ref + alt). Every heterozygote locus presented well balanced alleles with mean read frequency between 0.4 and 0.6, the commonly applied lower and upper thresholds [45,46,47], respectively (Figure 2b). Moreover, 75% of these loci showed an average value in the range of 0.5 ± 0.03. Only six loci had an allele read frequency in one of the replicates that transgressed the above thresholds for heterozygotes, three of them exceeding 0.6 (rs10483251, rs2503770 and rs7570971) and the remaining three below 0.4 (rs2337024, rs345769 and rs4792928). Allele read frequency was also estimated for the remaining amounts of input DNA of the dilution series (Supplementary Figure S1). The first markers out of the threshold range appeared at 0.125 ng, showing that heterozygote SNPs remain well balanced even at low DNA inputs.

The accuracy of the VISAGE BT A&A (PSeq) assay to detect the correct nucleotide at the position of the SNPs of interest was also studied by calculating base misincorporation percentages [45]. Specific base misincorporation (SBM) was inferred from loci with homozygote genotypes, where erroneously incorporated nucleotides matching the possible alternative allele can be observed. Non-specific base misincorporation (NSM), produced by the incorporation of nucleotides different to those of the possible alleles, was calculated from every SNP. Figure 2c shows the distribution of the mean misincorporation percentage of each SNP. The average total misincorporation percentage of the VISAGE BT A&A (PSeq) was of 0.14% per SNP, with 0.09% of SBM and 0.05% of NSM. Six loci were free of any type of misincorporation. In total, 31.4% of the markers, presented a misincorporation percentage higher than the average, however, only six loci were over 0.3%. SNP rs1393350 showed the highest misincorporation rate observed, 0.54% (0.50% SBM and 0.04% NSM). This was produced by one of the replicates that had 1.4% of alternative allele (adenine) reads for this SNP, while it stayed below 0.07% in the remaining replicates. The visual examination revealed that this misincorporation could be due to the presence of a small polynucleotide tract of three adenines located downstream of the SNP (Supplementary Figure S2). This poly-A tract may mislead the polymerase, incorporating an adenine instead of the real nucleotide of the SNP. Nevertheless, as the result of the three replicates showed, the misincorporation observed in this SNP is a sporadic event, due to a stochastic effect not associated with the VISAGE BT A&A (PSeq) assay.

### 3.5. Casework “Mock” Samples

The application of the VISAGE BT A&A (PSeq) assay to five casework-type DNA samples from GEDNAP resulted in 764 genotype calls with only one locus drop-out (no call) observed in one of the samples. This “mock” sample, 44-S3, was also the one with the highest number of markers below 200 reads, 6.54% (10 SNPs), even though it ranked second by number of total reads (Table 1).

These casework-type samples showed mean reads per marker values similar to those obtained for the replicates of 0.05 ng of control DNA analyzed for the sensitivity test, which had 10-fold less template DNA. This may be due to the nature of the DNA extracted from the casework-type samples, which generally had undergone degradation, and to the long time that some samples were stored in freezers. In this sense, these results were in agreement with previous studies where the SNP genotyping results from artificially degraded samples were comparable to the results obtained from non-degraded samples with much lower DNA amounts, depending on the level of degradation [39,48].

### 3.6. Concordance

Genotyping concordance of the results obtained with the VISAGE BT A&A (PSeq) assay was tested analyzing four Coriell DNA samples with known genotypes that are published in databases of 1000 genomes [25,26] and SGDP [27]. All the SNPs included in BT A&A (PSeq) provided a full genotype in all four samples. Sample NA11200 lacked information for 3 SNPs in the database. From the 609 genotypes compared to the published data to check the concordance and genotyping accuracy of this panel, only one allele drop-out was observed (99.84% of concordance). This discrepancy was found in sample NA18498 for rs2789823, which should be heterozygous (AG), but resulted in a homozygous genotype (GG) with this panel. A visual examination of the sequence did not reveal ambiguities that could explain this result (Supplementary Figure S3). The flanking sequence of this sample appears to be free of any variation that could affect the correct hybridization of the primers. Thus, the discrepancy may be due to the sample itself. This was also observed in a previous analysis of an aliquot of the same sample performed with a different assay for VISAGE BT A&A based on AmpliSeq chemistry and using Ion Torrent S5 as MPS platform [23]. Notably, the VISAGE BT A&A (Amp) assay, which used independently designed primers, different amplification chemistry, different MPS chemistry and different MPS technology, obtained the same homozygous GG genotype at rs2789823 for sample NA18498 as observed here with the VISAGE BT A&A (PSeq) assay, reinforcing the inference that the discrepancy could be due to the sample itself. This is not unusual in DNA samples extracted from cell lines that are the product of subsequent culturing, which could introduce some genotype shift [49,50]. Nevertheless, in a new 1000 Genome high-coverage dataset produced at the New York Genome Center [25], sample NA18498 has the homozygous GG genotype at rs2789823. Hence, the heterozygous AG genotype observed at the 1000 Genomes Phase 3 database [26] could be erroneous and instead the genotype obtained with this VISAGE BT A&A (PSeq) assay would be correct.

## 4. Conclusions

This study describes the evaluation of the VISAGE BT A&A (PSeq) assay that includes 153 DNA markers for simultaneous eye, hair, and skin color as well as continental BGA prediction. This assay has demonstrated it produces reliable and concordant results when control DNA and Coriell samples are analyzed using 0.5 ng. The VISAGE BT A&A (PSeq) assay also gave complete genotypes at all SNPs down to 125 pg, and 99.67% of the markers were correctly genotyped with 50 pg, confirming its suitability for forensic sample amounts. This VISAGE BT A&A (PSeq) assay provides well-balanced results in most of the markers. It also presented very low baseline noise and low misincorporation in the majority of the SNPs. The analysis of casework-type samples shows promising results and suggests that this tool can be usefully applied to forensic genetics. This study expands the applicability of the VISAGE Basic Tool for Appearance and Ancestry to both forensically used MPS platforms, the Ion S5 [23] and the Illumina Miseq FGx.

## Figures and Tables

**Table 1:** Summary statistics of number of reads obtained with the VISAGE BT A&A (PSeq) assay for casework-type samples. “Mean per SNP” indicates the average read depth per single nucleotide polymorphism (SNP) and “SD per SNP” is the standard deviation.

Sample	42-S3	44-S3	45-S2	49-S4	53-S1
Total reads	329,220	406,615	492,074	361,511	260,588
Mean per SNP	2151	2657	3216	2362	1703
SD per SNP	1351	2384	2104	1491	1202
SNPs with < 200 reads	2	11	2	3	9

## Supplementary Materials

The following are available online at https://www.mdpi.com/2073-4425/11/6/708/s1, Figure S1: Distribution of the allele read frequency of heterozygote loci of every 2800 M Control DNA dilution. A continuous line marks the expected optimum (0.5) while the dotted lines indicate the lower and upper thresholds (0.4 and 0.6, respectively). (a) Allele read frequency of replicates with 1 ng DNA. (b) Allele read frequency of replicates with 0.25 ng DNA. (c) Allele read frequency of replicates with 0.125 ng DNA. (d) Allele read frequency of replicates with 0.05 ng DNA. (e) Allele read frequency of replicates with 0.01 ng DNA, Figure S2: Visualization of sequence alignment of the three replicates of 0.5 ng for rs1393350 with IGV from results obtained with the VISAGE BT A&A (PSeq) assay, Figure S3: Visualization of sequence alignment of sample NA18498 for rs2789823 with IGV from results obtained with the VISAGE BT A&A (PSeq) assay.

## Appendix A

Centres and investigators of the VISible Attributes through GEnomics (VISAGE) Consortium

**Website:** **http://www.visage-h2020.eu/**

- **Erasmus University Medical Center Rotterdam, Rotterdam, The Netherlands:** Manfred Kayser, Vivian Kalamara, Arwin Ralf, Athina Vidaki
- **Jagiellonian University, Krakow, Poland:** Wojciech Branicki, Ewelina Pośpiech, Aleksandra Pisarek
- **Universidade de Santiago de Compostela, Santiago de Compostela, Spain :** Ángel Carracedo, Maria Victoria Lareu, Christopher Phillips, Ana Freire-Aradas, Ana Mosquera-Miguel, María de la Puente
- **Medizinische Universität Innsbruck, Innsbruck, Austria:** Walther Parson, Catarina Xavier, Antonia Heidegger, Harald Niederstätter
- **Universität zu Köln, Cologne, Germany:** Michael Nothnagel, Maria-Alexandra Katsara, Tarek Khellaf
- **King’s College London, London, UK:** Barbara Prainsack, Gabrielle Samuel
- **Klinikum der Universität zu Köln, Cologne, Germany:** Peter M. Schneider, Theresa E. Gross, Jan Fleckhaus
- **Bundeskriminalamt, Wiesbaden, Germany:** Ingo Bastisch, Nathalie Schury, Jens Teodoridis, Martina Unterländer
- **Institut National De Police Scientifique, Lyon, France:** François-Xavier Laurent, Caroline Bouakaze, Yann Chantrel, Anna Delest, Clémence Hollard, Ayhan Ulus, Julien Vannier
- **Netherlands Forensic Institute, The Hague, Netherlands:** Titia Sijen, Kris van der Gaag, Marina Ventayol-Garcia
- **National Forensic Centre, Swedish Police Authority, Linköping, Sweden:** Johannes Hedman, Klara Junker, Maja Sidstedt
- **Metropolitan Police Service, London, United Kingdom:** Shazia Khan, Carole E. Ames, Andrew Revoir
- **Centralne Laboratorium Kryminalistyczne Policji, Warsaw, Poland:** Magdalena Spólnicka, Ewa Kartasinska, Anna Woźniak

## References

[1] Kayser M. (2015). Forensic DNA Phenotyping: Predicting human appearance from crime scene material for investigative purposes. Forensic Sci. Int. Genet..

[2] Phillips C. (2015). Forensic genetic analysis of bio-geographical ancestry. Forensic Sci. Int. Genet..

[3] Schneider P.M., Prainsack B., Kayser M. (2019). The Use of Forensic DNA Phenotyping in Predicting Appearance and Biogeographic Ancestry. Dtsch. Arztebl. Int..

[4] Butler K., Peck M., Hart J., Schanfield M., Podini D. (2011). Molecular “eyewitness”: Forensic prediction of phenotype and ancestry. Forensic Sci. Int. Genet. Suppl. Ser..

[5] Walsh S., Chaitanya L., Breslin K., Muralidharan C., Bronikowska A., Pospiech E., Koller J., Kovatsi L., Wollstein A., Branicki W. (2017). Global skin colour prediction from DNA. Hum. Genet..

[6] Hernando B., Ibanez M.V., Deserio-Cuesta J.A., Soria-Navarro R., Vilar-Sastre I., Martinez-Cadenas C. (2018). Genetic determinants of freckle occurrence in the Spanish population: Towards ephelides prediction from human DNA samples. Forensic Sci. Int. Genet..

[7] Hysi P.G., Valdes A.M., Liu F., Furlotte N.A., Evans D.M., Bataille V., Visconti A., Hemani G., McMahon G., Ring S.M. (2018). Genome-wide association meta-analysis of individuals of European ancestry identifies new loci explaining a substantial fraction of hair color variation and heritability. Nat. Genet..

[8] Pospiech E., Chen Y., Kukla-Bartoszek M., Breslin K., Aliferi A., Andersen J.D., Ballard D., Chaitanya L., Freire-Aradas A., van der Gaag K.J. (2018). Towards broadening Forensic DNA Phenotyping beyond pigmentation: Improving the prediction of head hair shape from DNA. Forensic Sci. Int. Genet..

[9] Jing X., Sun Y., Zhao W., Gao X., Ma M., Liu F., Li C. (2019). Predicting adult height from DNA variants in a European-Asian admixed population. Int. J. Legal Med..

[10] Kukla-Bartoszek M., Pospiech E., Wozniak A., Boron M., Karlowska-Pik J., Teisseyre P., Zubanska M., Bronikowska A., Grzybowski T., Ploski R. (2019). DNA-based predictive models for the presence of freckles. Forensic Sci. Int. Genet..

[11] Liu F., Zhong K., Jing X., Uitterlinden A.G., Hendriks A.E.J., Drop S.L.S., Kayser M. (2019). Update on the predictability of tall stature from DNA markers in Europeans. Forensic Sci. Int. Genet..

[12] Walsh S., Liu F., Ballantyne K.N., van Oven M., Lao O., Kayser M. (2011). IrisPlex: A sensitive DNA tool for accurate prediction of blue and brown eye colour in the absence of ancestry information. Forensic Sci. Int. Genet..

[13] Walsh S., Liu F., Wollstein A., Kovatsi L., Ralf A., Kosiniak-Kamysz A., Branicki W., Kayser M. (2013). The HIrisPlex system for simultaneous prediction of hair and eye colour from DNA. Forensic Sci. Int. Genet..

[14] Chaitanya L., Breslin K., Zuniga S., Wirken L., Pospiech E., Kukla-Bartoszek M., Sijen T., Knijff P., Liu F., Branicki W. (2018). The HIrisPlex-S system for eye, hair and skin colour prediction from DNA: Introduction and forensic developmental validation. Forensic Sci. Int. Genet..

[15] Phillips C., Salas A., Sanchez J.J., Fondevila M., Gomez-Tato A., Alvarez-Dios J., Calaza M., de Cal M.C., Ballard D., Lareu M.V. (2007). Inferring ancestral origin using a single multiplex assay of ancestry-informative marker SNPs. Forensic Sci. Int. Genet..

[16] Kersbergen P., van Duijn K., Kloosterman A.D., den Dunnen J.T., Kayser M., de Knijff P. (2009). Developing a set of ancestry-sensitive DNA markers reflecting continental origins of humans. BMC Genet..

[17] Phillips C., Freire Aradas A., Kriegel A.K., Fondevila M., Bulbul O., Santos C., Serrulla Rech F., Perez Carceles M.D., Carracedo A., Schneider P.M. (2013). Eurasiaplex: A forensic SNP assay for differentiating European and South Asian ancestries. Forensic Sci. Int. Genet..

[18] Kidd K.K., Speed W.C., Pakstis A.J., Furtado M.R., Fang R., Madbouly A., Maiers M., Middha M., Friedlaender F.R., Kidd J.R. (2014). Progress toward an efficient panel of SNPs for ancestry inference. Forensic Sci. Int. Genet..

[19] Pereira V., Freire-Aradas A., Ballard D., Borsting C., Diez V., Pruszkowska-Przybylska P., Ribeiro J., Achakzai N.M., Aliferi A., Bulbul O. (2019). Development and validation of the EUROFORGEN NAME (North African and Middle Eastern) ancestry panel. Forensic Sci. Int. Genet..

[20] Eduardoff M., Gross T.E., Santos C., de la Puente M., Ballard D., Strobl C., Borsting C., Morling N., Fusco L., Hussing C. (2016). Inter-laboratory evaluation of the EUROFORGEN Global ancestry-informative SNP panel by massively parallel sequencing using the Ion PGM. Forensic Sci. Int. Genet..

[21] Mehta B., Daniel R., Phillips C., Doyle S., Elvidge G., McNevin D. (2016). Massively parallel sequencing of customised forensically informative SNP panels on the MiSeq. Electrophoresis.

[22] Breslin K., Wills B., Ralf A., Ventayol Garcia M., Kukla-Bartoszek M., Pospiech E., Freire-Aradas A., Xavier C., Ingold S., de La Puente M. (2019). HIrisPlex-S system for eye, hair, and skin color prediction from DNA: Massively parallel sequencing solutions for two common forensically used platforms. Forensic Sci. Int. Genet..

[23] Xavier C., de la Puente M., Mosquera-Miguel A., Freire-Aradas A., Kalamara V., Vidaki A., Gross T., Revoir A., Pośpiech E., Kartasińska E. (2020). Development and validation of the VISAGE AmpliSeq Basic Tool to predict appearance and ancestry from DNA. Forensic Sci. Int. Genet.

[24] Niederstätter H., Köchl S., Grubwieser P., Pavlic M., Steinlechner M., Parson W. (2007). A modular real-time PCR concept for determining the quantity and quality of human nuclear and mitochondrial DNA. Forensic Sci. Int. Genet..

[25] Consortium G.P. 1000 Genomes Project New York Genome Center High Coverage Dataset. http://ftp.1000genomes.ebi.ac.uk/vol1/ftp/data_collections/1000G_2504_high_coverage/working/20190425_NYGC_GATK/.

[26] Auton A., Brooks L.D., Durbin R.M., Garrison E.P., Kang H.M., Korbel J.O., Marchini J.L., McCarthy S., McVean G.A., Abecasis G.R. (2015). A global reference for human genetic variation. Nature.

[27] Mallick S., Li H., Lipson M., Mathieson I., Gymrek M., Racimo F., Zhao M., Chennagiri N., Nordenfelt S., Tandon A. (2016). The Simons Genome Diversity Project: 300 genomes from 142 diverse populations. Nature.

[28] Promega (2019). Prototype PowerSeq SNP System (March 2019).

[29] Promega (2018). ProNex Size-Selective Purification System. Technical Manual. TM508-2/18.

[30] Biosystems K. (2017). KAPA Hyper Prep Kit. Technical Data Sheet. KR0961-v6.1.

[31] Promega (2018). PowerSeq Quant MS System. Technical Manual. TM511-3/18.

[32] Li H., Durbin R. (2009). Fast and accurate short read alignment with Burrows-Wheeler transform. Bioinformatics.

[33] Li H., Handsaker B., Wysoker A., Fennell T., Ruan J., Homer N., Marth G., Abecasis G., Durbin R. (2009). The Sequence Alignment/Map format and SAMtools. Bioinformatics.

[34] McKenna A., Hanna M., Banks E., Sivachenko A., Cibulskis K., Kernytsky A., Garimella K., Altshuler D., Gabriel S., Daly M. (2010). The Genome Analysis Toolkit: A MapReduce framework for analyzing next-generation DNA sequencing data. Genome Res..

[35] Van der Auwera G.A., Carneiro M.O., Hartl C., Poplin R., Del Angel G., Levy-Moonshine A., Jordan T., Shakir K., Roazen D., Thibault J. (2013). From FastQ data to high confidence variant calls: The Genome Analysis Toolkit best practices pipeline. Curr. Protoc. Bioinform..

[36] Robinson J.T., Thorvaldsdottir H., Wenger A.M., Zehir A., Mesirov J.P. (2017). Variant Review with the Integrative Genomics Viewer. Cancer Res..

[37] Team R.C. (2019). R: A Language and Environment for Statistical Computing.

[38] RStudio (2018). RStudio: Integrated Development for R.

[39] Jager A.C., Alvarez M.L., Davis C.P., Guzman E., Han Y., Way L., Walichiewicz P., Silva D., Pham N., Caves G. (2017). Developmental validation of the MiSeq FGx Forensic Genomics System for Targeted Next Generation Sequencing in Forensic DNA Casework and Database Laboratories. Forensic Sci. Int. Genet..

[40] Avent I., Kinnane A.G., Jones N., Petermann I., Daniel R., Gahan M.E., McNevin D. (2019). The QIAGEN 140-locus single-nucleotide polymorphism (SNP) panel for forensic identification using massively parallel sequencing (MPS): An evaluation and a direct-to-PCR trial. Int. J. Legal Med..

[41] de la Puente M., Phillips C., Xavier C., Amigo J., Carracedo A., Parson W., Lareu M.V. (2020). Building a custom large-scale panel of novel microhaplotypes for forensic identification using MiSeq and Ion S5 massively parallel sequencing systems. Forensic Sci. Int. Genet..

[42] Budowle B., Eisenberg A.J., van Daal A. (2009). Validity of low copy number typing and applications to forensic science. Croat. Med. J..

[43] Gill P., Haned H., Bleka O., Hansson O., Dorum G., Egeland T. (2015). Genotyping and interpretation of STR-DNA: Low-template, mixtures and database matches-Twenty years of research and development. Forensic Sci. Int. Genet..

[44] Buchard A., Kampmann M.L., Poulsen L., Borsting C., Morling N. (2016). ISO 17025 validation of a next-generation sequencing assay for relationship testing. Electrophoresis.

[45] Eduardoff M., Santos C., de la Puente M., Gross T.E., Fondevila M., Strobl C., Sobrino B., Ballard D., Schneider P.M., Carracedo A. (2015). Inter-laboratory evaluation of SNP-based forensic identification by massively parallel sequencing using the Ion PGM. Forensic Sci. Int. Genet..

[46] Grandell I., Samara R., Tillmar A.O. (2016). A SNP panel for identity and kinship testing using massive parallel sequencing. Int. J. Legal Med..

[47] Guo F., Yu J., Zhang L., Li J. (2017). Massively parallel sequencing of forensic STRs and SNPs using the Illumina^®^ ForenSeq DNA Signature Prep Kit on the MiSeq FGx Forensic Genomics System. Forensic Sci. Int. Genet..

[48] Fattorini P., Previdere C., Carboni I., Marrubini G., Sorcaburu-Cigliero S., Grignani P., Bertoglio B., Vatta P., Ricci U. (2017). Performance of the ForenSeq(TM) DNA Signature Prep kit on highly degraded samples. Electrophoresis.

[49] Schafer C.M., Campbell N.G., Cai G., Yu F., Makarov V., Yoon S., Daly M.J., Gibbs R.A., Schellenberg G.D., Devlin B. (2013). Whole exome sequencing reveals minimal differences between cell line and whole blood derived DNA. Genomics.

[50] International Cell Line Authentication Committee (ICLAC) (2014). Guide to Human Cell Line Authentication. http://iclac.org/wp-content/uploads/Authentication-SOP_09-Jan-2014.pdf.

