# Supplementary material for: Evaluation of the VISAGE Basic Tool for Appearance and Ancestry Prediction Using PowerSeq Chemistry on the MiSeq FGx System
Source: Genes (Basel). 2020 Jun 26;11(6):708. doi: 10.3390/genes11060708 (PMC7349024; doi:10.3390/genes11060708)
Supplement: Supplementary file 1 [file genes-11-00708-s001.pdf]

(a)

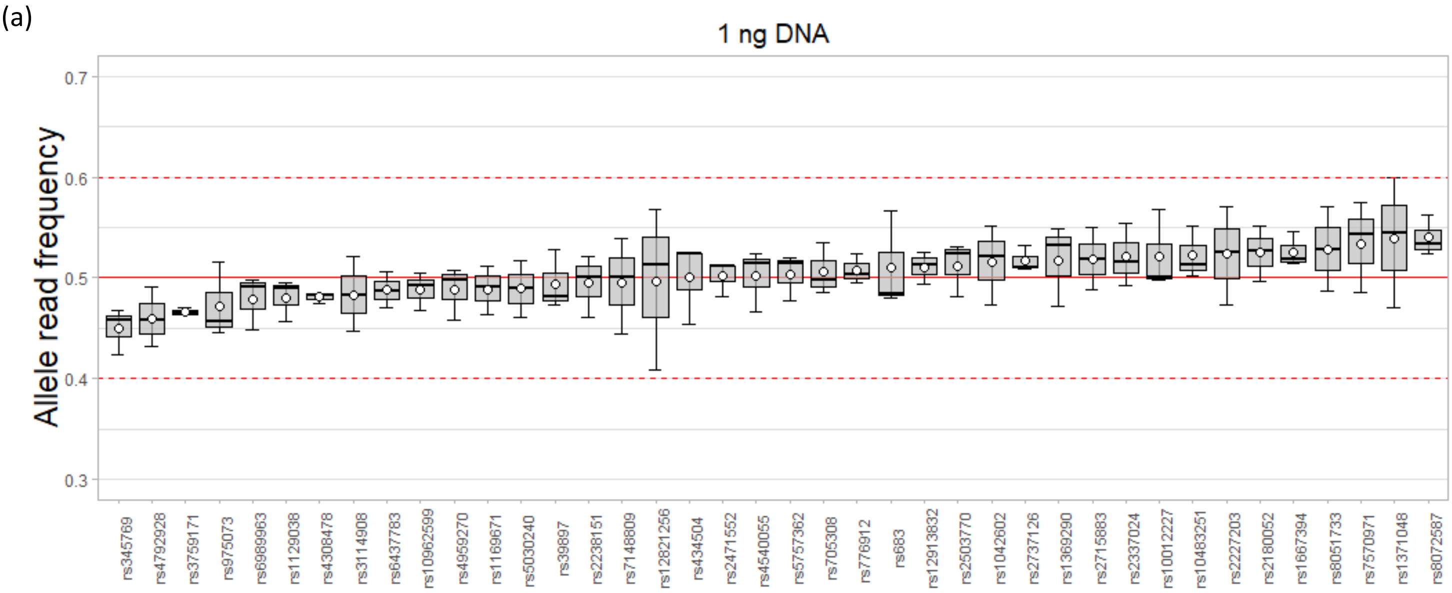

(b)

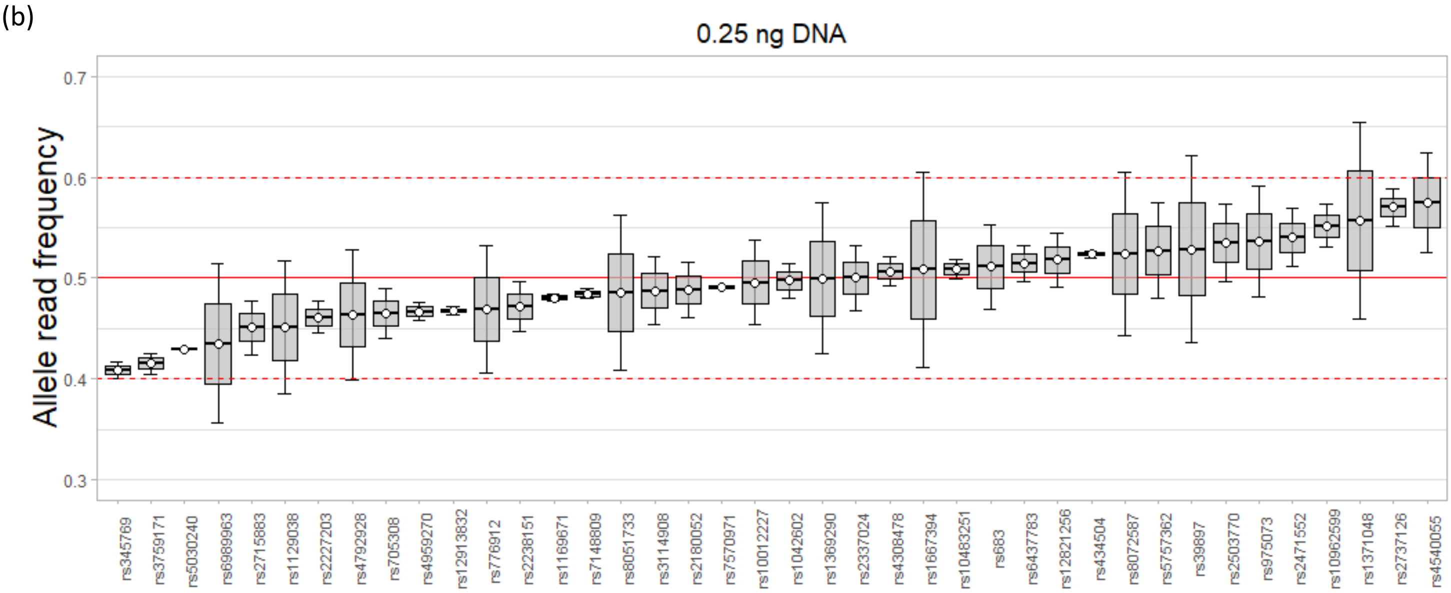

(c)

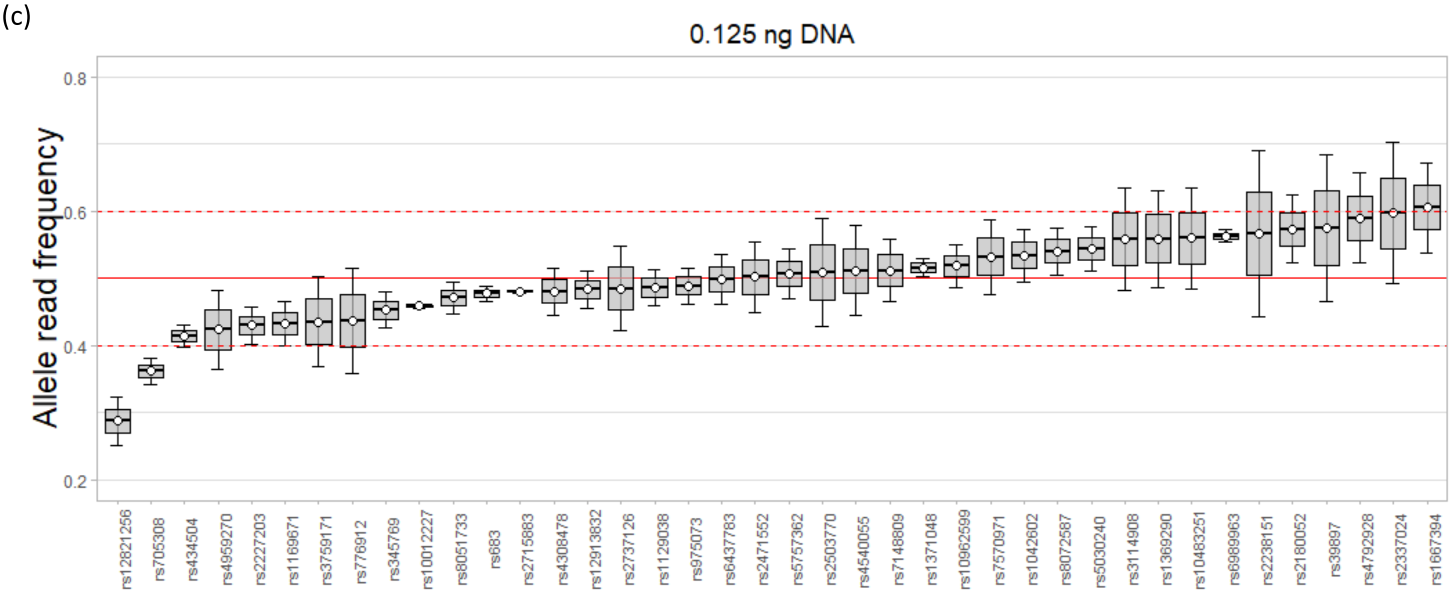

Figure S1. Distribution of the allele read frequency of heterozygote loci of every 2800 M Control DNA dilution. A continuous line marks the expected optimum (0.5) while the dotted lines indicate the lower and upper thresholds (0.4 and 0.6, respectively). (a) Allele read frequency of replicates with 1 ng DNA. (b) Allele read frequency of replicates with 0.25 ng DNA. (c) Allele read frequency of replicates with 0.125 ng DNA. (d) Allele read frequency of replicates with 0.05 ng DNA. (e) Allele read frequency of replicates with 0.01 ng DNA.

(d)

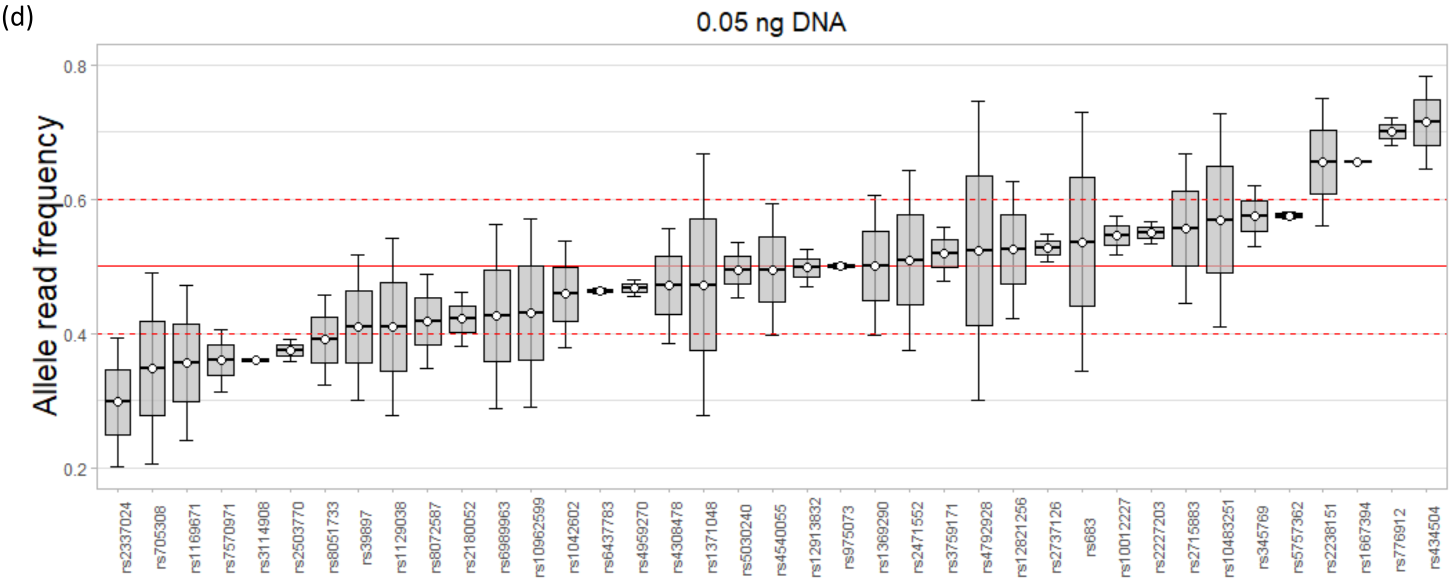

(e)

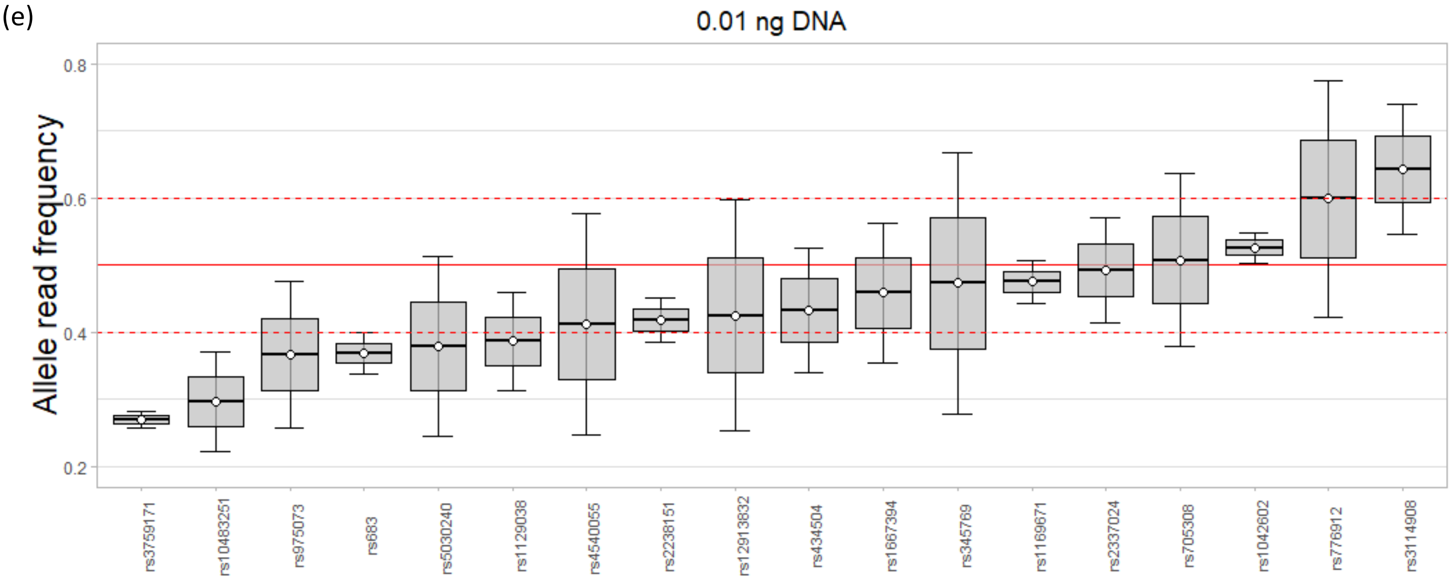

Figure S1. *Continuation*.

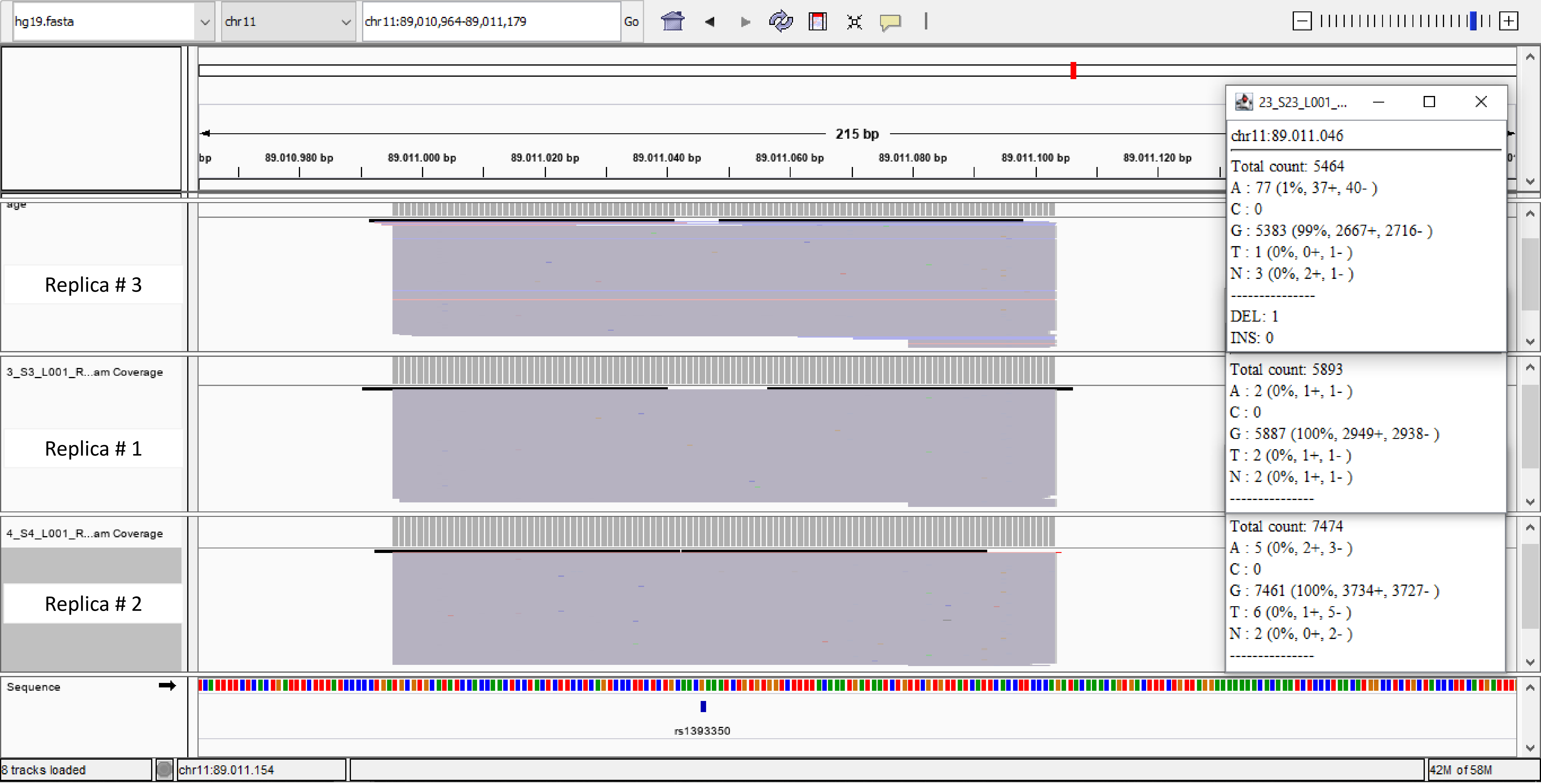

Figure S2. Visualization of sequence alignment of the three replicates of 0.5 ng for rs1393350 with IGV from results obtained with the VISAGE BT A&A (PSeq) assay.

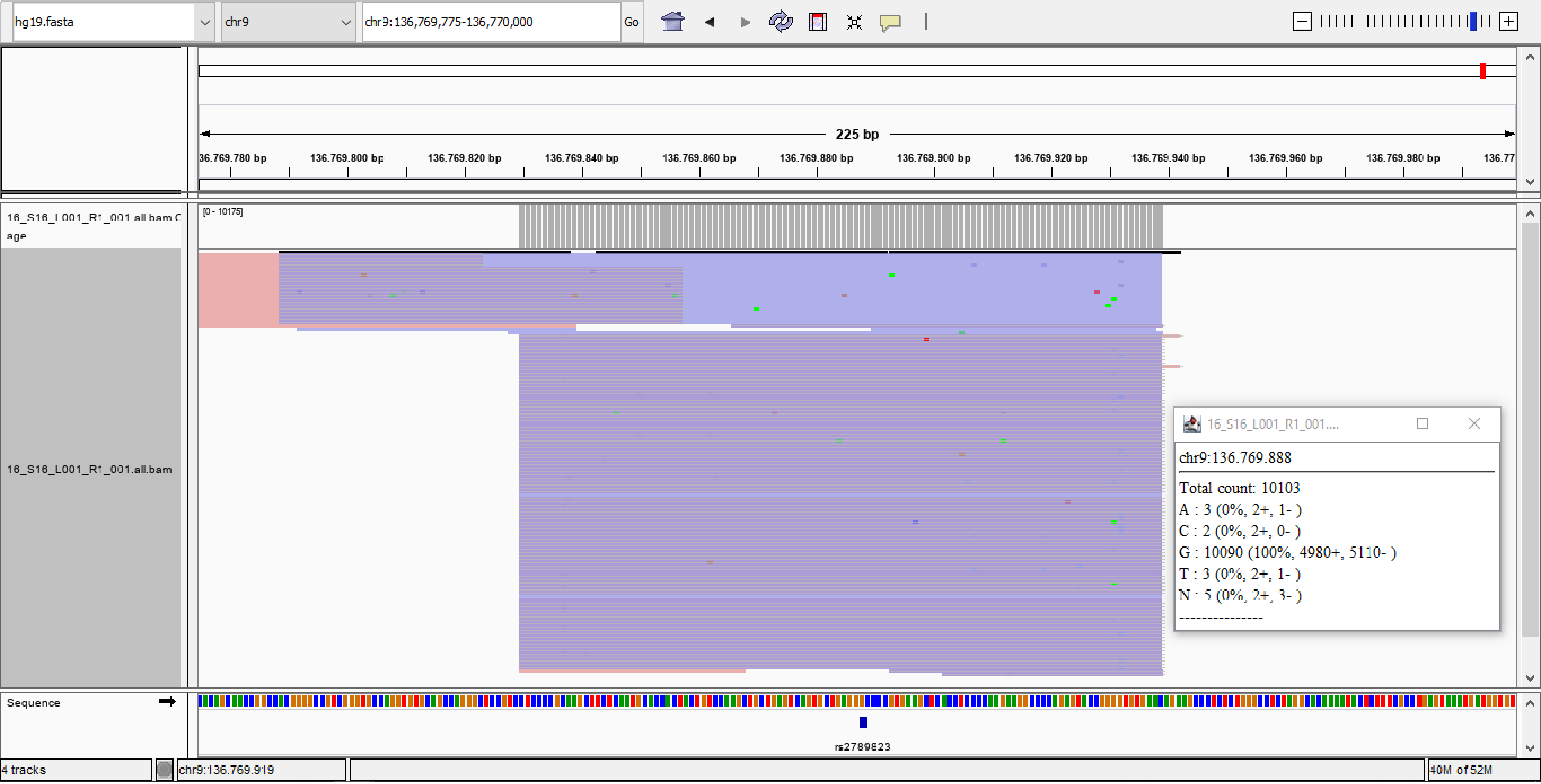

Figure S3. Visualization of sequence alignment of sample NA18498 for rs2789823 with IGV from results obtained with the VISAGE BT A&A (PSeq) assay.
